# Integrative approach of omics and imaging data to discover new insights for understanding brain diseases

**DOI:** 10.1093/braincomms/fcae265

**Published:** 2024-08-08

**Authors:** Jong Hyuk Yoon, Hagyeong Lee, Dayoung Kwon, Dongha Lee, Seulah Lee, Eunji Cho, Jaehoon Kim, Dayea Kim

**Affiliations:** Neurodegenerative Diseases Research Group, Korea Brain Research Institute, Daegu 41062, Republic of Korea; Neurodegenerative Diseases Research Group, Korea Brain Research Institute, Daegu 41062, Republic of Korea; Neurodegenerative Diseases Research Group, Korea Brain Research Institute, Daegu 41062, Republic of Korea; Cognitive Science Research Group, Korea Brain Research Institute, Daegu 41062, Republic of Korea; Neurodegenerative Diseases Research Group, Korea Brain Research Institute, Daegu 41062, Republic of Korea; Neurodegenerative Diseases Research Group, Korea Brain Research Institute, Daegu 41062, Republic of Korea; Neurodegenerative Diseases Research Group, Korea Brain Research Institute, Daegu 41062, Republic of Korea; New Drug Development Center, Daegu-Gyeongbuk Medical Innovation Foundation (K-MEDI hub), Daegu 41061, Republic of Korea

**Keywords:** brain diseases, genomics, MRI, PET, proteomics

## Abstract

Treatments that can completely resolve brain diseases have yet to be discovered. Omics is a novel technology that allows researchers to understand the molecular pathways underlying brain diseases. Multiple omics, including genomics, transcriptomics and proteomics, and brain imaging technologies, such as MRI, PET and EEG, have contributed to brain disease-related therapeutic target detection. However, new treatment discovery remains challenging. We focused on establishing brain multi-molecular maps using an integrative approach of omics and imaging to provide insights into brain disease diagnosis and treatment. This approach requires precise data collection using omics and imaging technologies, data processing and normalization. Incorporating a brain molecular map with the advanced technologies through artificial intelligence will help establish a system for brain disease diagnosis and treatment through regulation at the molecular level.

## Teaser

Yoon *et al*. suggest that establishing brain multi-molecular maps using an artificial intelligence-based integrative approach of omics and imaging will provide new insights at the molecular level into diagnosis and treatment that are more accurate than the currently available therapies.

## Graphical Abstract

**Graphical abstract:**
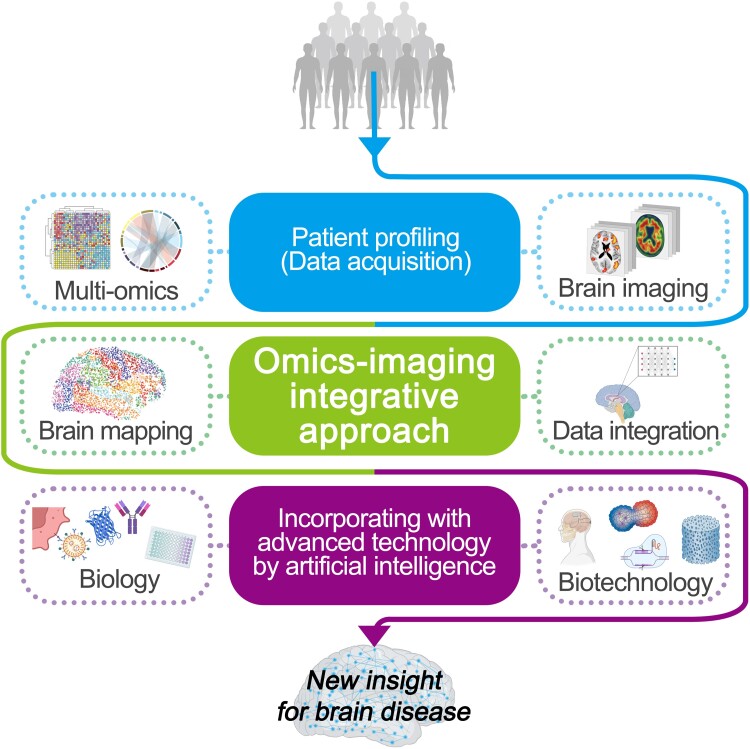

## Introduction

The brain, at the centre of the nervous system, is a key organ that controls each part of the body.^1^ There are considerable challenges in the development and improvement of therapeutic strategies for brain diseases because of the unique characteristics of the brain (Box 1).^1-4^ For example, the development of treatments for brain diseases is complicated by the restricting capacity of the blood–brain barrier, which maintains CNS homeostasis and protects the brain from infection and toxin exposure by regulating the passage of various substances into the blood.^5^ The blood–brain barrier impedes sufficient entry of certain drugs and large-molecule therapeutics into the brain for effective pharmacological treatment.^5,6^ Recently, nanoscale brain targeting strategies have evolved via invasive or non-invasive routes; however, the clinical applications of these strategies have not yet been fully established.^5,6^ Furthermore, even if a method for the delivery of therapeutic drugs to the desired brain region is devised, damaged nerve cells of the CNS cannot revert to the pre-damaged state.^3,7^ For example, in Alzheimer's disease, no therapy exists for the treatment or reversal of brain damage, such as brain shrinkage and synaptic loss, occurring in multiple brain regions and observable using MRI.^8^ Moreover, most brain pathologies are not triggered by a single element but are caused by complex molecular mechanisms.^2,3^ Brain diseases are a heterogeneous group of diseases with multiple aetiologies; complex underlying mechanisms including pathological protein aggregation, neuronal and synaptic network dysfunction, altered energy homeostasis, nucleic acid defects, neuroinflammation and neuronal cell death; and a range of clinical features including social impairment.^3,9,10^ Furthermore, animal models used to probe diseases related to the brain exhibit limited disease mimicry and cannot completely reproduce the clinical conditions observed in humans.^2,4,11^ However, conducting such experiments using living human brains remains ethically controversial. Despite these difficulties, brain research has attracted more attention owing to the severity of clinical symptoms such as cognitive impairment and behavioural disturbances as the disease progresses over time.^12-15^ A variety of advanced technologies have been used to investigate the mechanisms underlying brain pathology to identify therapeutic approaches to alleviate disease progression.

Box 1 **Challenges in the development of effective treatments for brain diseases**

- The blood–brain barrier hinders the uptake of many types of therapeutic materials to the brain.
- Recovery of damaged brain cells in the CNS is limited.
- Most brain diseases are caused by complex molecular pathologies rather than a specific single factor.
- Reproducing exact clinical conditions in animal models is difficult.
- Ethical issues arise with experiments involving the live human brain.

Omics is a high-throughput technology that has flourished in biomedical research by allowing the simultaneous analysis of a wide range of biological molecules.^16^ In brain research, omics has contributed to the comprehensive understanding and mapping of molecular networks associated with brain diseases, including Alzheimer's disease, Parkinson's disease, autism spectrum disorder and stroke.^16-19^ Using brain tissues and biofluids, such as CSF or blood, omics profiling with genomics, transcriptomics, proteomics and metabolomics has identified systemic molecular variations in the pathological brain.^16-19^ Now that a clear cause and treatment for various dysfunctions in the brain have not been suggested, there is a growing demand for developing biomarkers to aid the early diagnosis of brain diseases and elucidate the precise molecular mechanisms underlying the pathological conditions.^20^ Omics approaches also play a crucial role in advancing biomarkers by analysing omics data from multiple layers.^21-24^ Identification of molecular biomarkers through integrated analysis of omics profiles, known as multi-omics, provides powerful insights into the diagnosis, treatment and prevention of brain diseases.^25-27^

Psychoneurological examinations are performed for diagnostic purposes in the early stages of disease for a range of brain diseases including brain cancers, cerebrovascular diseases, psychiatric disorders and neurodegenerative diseases; however, the most widely used investigation is brain imaging.^28-32^ Because the brain is a physiologically dynamic organ, brain imaging technologies capable of visualizing the active brain at high resolution are essential to precisely detect abnormalities.^33^ Brain imaging technologies are a non-invasive approach allowing the visualization of the structure and understanding of the function of the brain. Structural and functional evaluation of the brain can identify brain damage and establish the relationship between specific regions of the brain and their functions.^34,35^ Structural evaluation is performed using MRI, CT and cerebral angiography, whereas functional evaluation is performed using PET, single photon emission CT, functional MRI (fMRI), EEG and magnetoencephalography.^33^ Recently, the development of imaging biomarkers with sufficient sensitivity and specificity to be used in clinical practice has been reported.^36,37^ To develop more effective strategies for the diagnosis and treatment of brain diseases, highly reliable biomarkers are needed.

Advances in multi-omics, including metabolomics, proteomics, transcriptomics, genomics and brain imaging techniques, have enabled the development of functional biomarkers and personalized medicines at a highly detailed molecular level.^38^ Biomarkers have strong potential for use in disease diagnosis, classification and prognostic prediction, particularly in the early and pre-onset stages of the disease.^39^ Accordingly, these technologies have contributed to the identification of therapeutic targets in clinical trials.^39^ However, individual technologies cannot fully capture the biological complexity of human brain diseases.^40^ We aimed to combine multi-omics with imaging datasets to propose novel approaches for understanding brain pathology.

## Omics approaches for brain diseases

### Genomics

Genomics is a relatively mature field and involves the study of the entire genome, focusing on genetic variations and their associated functions.^41^ The role of genomics in the aetiology of brain disease has enabled the identification of pathogenic genes.^41^ Genome-wide association studies are powerful tools that have led to the identification of genomic variants related to disease aetiology, such as single nucleotide variations and copy number variations.^42,43^ Next-generation sequencing technologies, whole-exome sequencing, whole-genome sequencing and targeted sequencing have also contributed to the detection of unusual mutations that are rarely identified using genome-wide association studies.^16^ The onset and progression of brain diseases that may or may not be inherited, depending on the type of disease, can be affected by abnormal changes in specific genes.^16^ Neurodegeneration in Alzheimer's disease and Parkinson's disease increases with age and can be triggered by the accumulation of abnormal proteins due to a wide range of genetic causes.^44^ Previous genomic studies have shown that rare mutations in *amyloid precursor protein*, *PSEN1* (*presenilin-1*), and *PSEN2* (*presenilin-2*) and polymorphisms in many genetic regions, including *apolipoprotein E* are involved in early-onset Alzheimer's disease and late-onset Alzheimer's disease, respectively.^16^ Additionally, variants in risk genes, including *alpha-synuclein*, *PARK16* (*Parkinson disease 16*), *LRRK2* (*leucine-rich repeat kinase 2*), *microtubule-associated protein tau* and *BST1* (*bone marrow stromal cell antigen 1*) have been identified as causal factors for Parkinson's disease.^45^ Furthermore, the neurodevelopmental symptoms of autism spectrum disorder that appear in early childhood are highly associated with inherited factors and *de novo* genetic alterations that have not been fully elucidated.^46^

### Transcriptomics

Transcriptomics investigates the transcriptome, which includes all the RNA transcripts produced by a cell.^41^ Transcriptomic analysis has been used to identify changes in gene expression and regulation by detecting key targets, such as differentially expressed genes, non-coding RNAs, alternative splicing and copy number variations related to the pathological mechanisms underlying brain diseases.^47^ To screen transcriptomes, RNA sequencing and microRNA microarray techniques are applied to analyse the targets of brain cells, tissues and several body fluids, such as blood or CSF.^47^ MicroRNAs are good examples of biofluid markers for Alzheimer's disease diagnosis in transcriptomic analyses because they play key roles in regulating brain functions.^48^ Dysregulation of microRNA expression has important implications for pathways related to Alzheimer's disease, including amyloid formation, amyloid precursor protein processing, tau phosphorylation and inflammation mechanism.^47^ Furthermore, a recent study combining spatial transcriptomics and in situ sequencing revealed that a plaque-induced gene network and oligodendrocyte gene response in mouse and human brains at the cellular level are hallmarks of Alzheimer's disease.^49^ Additionally, alterations of the prominent functional pathways in Parkinson's disease brain tissues are associated with dopamine metabolism, neuroinflammation, oxidative stress, mitochondrial function, protein degradation, vesicular transport and synaptic transmission.^50,51^ Transcriptomic approaches using blood samples have also revealed changes in Parkinson's disease-related gene expression, including pathways involved in immune function, inflammation, mitochondrial function, protein chaperones, RNA processing and programmed cell death.^50^

### Proteomics

Proteomics is the large-scale study of proteomes and the entire set of proteins expressed based on their genetic backgrounds. Proteomics analysis is widely used for the identification, quantification, conductance of interaction studies and modification of proteins.^41^ Importantly, this allows the analysis of post-translational modifications such as phosphorylation, ubiquitination, methylation, acetylation, sumoylation and glycosylation, which cannot be obtained through genomic analysis and complicate the functional diversity of proteins.^52^ The rapid development of proteomics is based on state-of-the-art technologies, particularly liquid chromatography-mass spectrometry (LC-MS). LC-MS-based methods have allowed high-throughput profiling of global proteins in brain and biofluids.^41,53^ In an analysis of the brain and CSF proteome using multiplex MS, classified protein targets showing distinct pathophysiological processes focused on alterations in synaptic, metabolic, glial-enriched myelination and immunity protein panels in the Alzheimer's disease brain and CSF, Alzheimer's disease heterogeneity was observed.^54^ In another CSF proteome profiling using MS-based proteomics, levels of osteomodulin, nerve growth factor inducible (VGF), cluster of differentiation 44, prolactin and mannosidase alpha class 2B member 1 were altered in patients with Parkinson's disease, correlating with the severity of Parkinson's disease pathology and levels of cathepsin S, phospholipase D4 and human leukocyte antigen were increased, indicating elevated neuroinflammation in LRRK2 G2019S carriers.^55^ Additionally, dysregulation of CSF arginine vasopressin is robustly associated with autism spectrum disorder diagnosis and social symptom severity, even at an early age, indicating that is a promising CSF marker of autism spectrum disorder.^56^ Moreover, some candidate proteins, mostly secreted proteins, have been identified as promising biomarkers and drug targets for stroke in tandem mass tag-coupled proteomic analysis of the mouse brain cortex.^57^

### Metabolomics

Metabolomics is the study of multiple small molecules (<1500 Da), such as amino acids, fatty acids, carbohydrates and other products of cellular metabolism.^41^ These metabolites are abundant in biofluids and can be influenced by alterations in genetics, transcripts, proteins and various environmental factors, and serve as energy sources, metabolic intermediates and signalling molecules.^16,58^ Methods for metabolomic analysis include Fourier-transform infrared spectroscopy, nuclear magnetic resonance spectroscopy and MS-based approaches, which are powerful tools for detecting a wide spectrum of metabolites.^58^ Additionally, the goals of metabolomics studies vary between targeted and untargeted analyses. Targeted approaches are mostly used to identify and quantify specific metabolites, whereas untargeted approaches aim to annotate metabolites and review extensive metabolic changes.^59^ Targeted metabolomics in the blood and brain in longitudinal cohorts revealed that baseline levels of serum metabolites, such as glycerophospholipids, amino acids and acylcarnitine, predict the risk of incident Alzheimer's disease and cognitive changes. It is indicated that acylcarnitine has significant association between central and peripheral metabolites with Alzheimer's disease phenotypes, but there were few connections between two organs according to partial correlation and network analyses.^60^ Additionally, LC-MS analysis revealed that the plasma metabolome of Parkinson's disease, which showed altered profiles in drug-naïve Parkinson's disease compared with the control, was influenced by L-dopa treatment and suggested potential biomarker candidates, including free fatty acid 12:0, free fatty acid 10:0, indolelactic acid and phenylacetyl-glutamine.^61^ Additionally, MS signal intensities of metabolites revealed differences in the prefrontal cortex grey matter between the autism spectrum disorder and control groups, providing the prospect of new instruments for autism spectrum disorder diagnosis and prediction.^62^ In the absence of a general agreement for metabolomic signatures in depression models, altered levels of metabolites in the brain, urine and blood samples of animal models were investigated.^63^ Metabolomic alterations were revealed by changes in the levels of neurotransmitters and kynurenine metabolites in the brain, amino acids and corticosterone in the blood, and microbial metabolites in the urine.^63^ Additionally, owing to the characteristics of metabolites, it is essential to simultaneously observe the expression and activity of proteins, which are units that produce and decompose metabolites.

### The multi-omics approach

As mentioned earlier, individual omics data provide a list of differences related to the disease.^41^ This information can be used to discover biomarkers of the disease and understanding the biological pathways that differ between the disease and healthy groups.^41^ However, utilizing only one type of data could give limited correlations like reactive processes, not causative ones.^41^ Furthermore, the results of individual omics studies were inadequate in identifying significant signals from the brain that are complex and dynamically changed. Thus, the biological complexity of brain disease has presented challenges to biologists, even with omics methods.^64^ Moreover, since the cause of most brain diseases is not simply a single risk factor, such as a specific gene or protein abnormality, previous genetic findings provide a limited understanding of diseases, such as imprecise mapping of causal variants and the gap between association and causation.^27^ Individual omics analysis likely assesses different views of the complex pathophysiology of complex pathogenesis and disease progression, and the analysis of just one omics subset gives a distorted, biased and imperfect view of the fundamental biology.^65^ Therefore, it is necessary to integrate the omics data for a broader perspective rather than utilizing individual omics data.^27^

Integration of individual omics data, called multi-omics, is used to explain the potential causative changes that induce diseases or the therapeutic targets that may be candidates for the subsequent molecular studies.^41^ Multi-omics allows a comprehensive understanding of complex traits associated with disease pathology. The development of this approach is gaining attention to illuminate disease aetiology and characterize new biomarkers for diseases.^66^ Multi-omics analysis using proteomic, transcriptomic and epigenomic profiling of postmortem human brains has shown that Alzheimer's disease involves the reconfiguration of the epigenome, wherein H3K9ac and H3K27ac affect Alzheimer's disease molecular pathways by disrupting transcription- and chromatin-gene feedback loops.^66^ The integration of single-nucleus RNA sequencing and phosphoproteome and proteome analysis revealed that *APOE4* gene induces an early disruption of the blood–brain barrier transcriptome in 2- to 3-month-old APOE4 knock-in mice.^67^ This results in dysregulation of the protein signal networks, which control cell junctions, clathrin-mediated transport, cytoskeleton and translation in brain endothelium, and transcription and RNA splicing suggestive of DNA damage in pericytes.^67^ Five-dimensional multi-omics analysis including proteomics, metabolomics, lipidomics and other factor profiling identified the difference within the cohort classified according to cognitive ability decline, and each dimension is differentially associated with core Alzheimer's disease pathology.^68^ In another case of multi-omics analysis comprising transcriptomics, proteomics and metabolomics, deregulated pathways related to vitamins, inflammation, neurotransmitter synapses, oxidative stress, complement and coagulation in Alzheimer's disease were observed.^69^ In human midbrains, integrative analyses of transcriptomics and proteomics identified Parkinson's disease-deregulated molecules, including *miR-539-3p, miR-376a-5p, miR-369-3p and miR-218-5p*, as well as proteins, such as heat shock protein family A member 1B, chitinase-3-like protein 1, folliculin-interacting protein 2 and tyrosine hydroxylase, suggesting that neuroinflammation, immune response and mitochondrial and synaptic dysfunction are therapeutic targets for advanced Parkinson's disease.^70^ By combining proteomic and metabolomic data derived from *Cntnap2*-knockout mouse cortex with omics data derived from patients with autism spectrum disorder, *Cntnap2*-associated autism spectrum disorder network models containing mitochondrial dysfunction, axonal impairment and synaptic activity have been established.^71^ Furthermore, the integration of genomics with plasma metabolomics using data-driven network analysis was performed in a cohort study to delineate major depressive disorder based on the age of onset, revealing differential biosignatures of major depressive disorder development.^72^ It is believed that multi-omics approaches give in-depth insight into brain diseases because multi-omics analysis includes multiple molecular profiling, metadata and big data processing with informatics and artificial intelligence (AI). Consequently, it provides new macroscopic and microscopic perspectives to understand brain diseases.^73^

### Limitations of omics-based approaches

The prevalence of brain disorders is increasing with the aging population. Individual omics technologies have systematically provided valuable information regarding the underlying mechanisms of diseases.^41^ However, the range of research conducted is limited by the specific molecular actions that can be detected using these tools.^74^ Genomics, which identifies genetic variations through genomic analysis, cannot identify the biological effects of epigenetic and post-translational changes.^74^ Transcriptomics, which profiles the pattern of gene expression and major pathways related to pathology, measures relative abundance that is not absolute but dynamic.^74^ Additionally, proteomics, which focuses on the detection of proteins that function in biological systems, yields different results owing to elution efficiency and post-translational modifications.^74,75^ Depending on data handling ability, extracting meaningful insights through the analysis of large-scale omics data from multiple sources, may not be feasible.^76^ Similar to other omics, for analysis based on a wide range of diverse targets, metabolomics relies on pre-processing and pre-treatment methods, sensitivity of analysis technology, global or targeted omics and researchers’ interpretation to produce significant outcomes.^74,77^ Furthermore, to map biomolecular information according to brain regions, it is necessary to obtain omics data using brain samples rather than biofluids, such as blood and CSF, with good accessibility to build large databases.

To understand the mechanisms of neuropathology, multi-omics data must be combined for data analysis, visualization and interpretation.^78^ With the accumulation of systematic multi-omics research, many different therapeutic candidates for brain diseases have been identified; however, strategies for understanding these diseases have not been completely elucidated.^79,80^ Furthermore, there are several challenges in multi-omics data integration, including missing values before statistical analysis, noise deletion, omics data heterogeneity and data normalization.^79,80^ As many trials using genome-wide association studies and multi-omics data to detect Alzheimer's disease risk genes and drug targets are not successful, attempts including drug repurposing have been made to apply statistics and machine/deep learning to multi-omics to gain new insights.^81^ However, despite many studies conducted, the data have not been robustly validated.^78,82^ Therefore, it is important to discuss the integration of macro and microscale approaches that make important contributions to the development of biomarkers for characterizing incurable brain diseases.

## Brain imaging

### MRI

MRI is a non-invasive technology for *in vivo* assessment that provides brain structural and functional information without radiation exposure.^83^ As protons in water molecules have a magnetic moment, MRI represents the internal structure of the human body by measuring the signals generated from the reaction of protons subjected to strong radiofrequency waves.^83^ Additionally, it is essential to map the connectome by visualizing neural connections to understand brain function because it works by forming a tight network between neurons. fMRI uses blood oxygenation level-dependent contrast to represent brain function by inferring which brain region is activated based on the level of oxygen consumption in the blood.^84^

MRI is widely used to detect multiple brain diseases.^85^ Recently, with the development of MRI, AI has also been used to predict specific brain abnormalities. Using computational resources based mainly on public MRI databases, AI was used to predict the survival of patients with brain tumours by focusing on the criteria of survival class and interval.^86^ In another study, the prognosis of mild cognitive impairment was predicted based on the patient's brain atrophy pattern in the hippocampus, identified through MRI data.^87^ Furthermore, research has been conducted to obtain the BigMac dataset by integrating macroscale MRI data of the macaque brain with microscale cellular organization using multi-contrast microscopy to understand the structural and functional information of the brain.^88^ Previous studies suggest the need not only to diagnose but also predict brain diseases that require early diagnosis using MRI technology and understand the pathological characteristics of the brain by combining other technologies rather than simply examining MRI data.

### PET

PET, like fMRI, does not directly evaluate neural activity, but indirectly measures neural activity by measuring changes in metabolism or blood flow due to the activation of local brain regions.^89^ PET localizes activated regions using a tracer that combines a radioactive isotope with a metabolite related to neuronal activation.^89,90^ The temporal resolution of the PET images is determined based on the half-lives of the isotopes used.^89-91^ For instance, PET can mirror the metabolic state of the human body using the glucose analogue called fluorine 18 fluorodeoxyglucose.^91^ This test is useful for detecting abnormal conditions involving hyperglycaemic glucose metabolism, such as Alzheimer's disease, malignant tumours, epilepsy and inflammatory diseases.^91-94^ PET has a relatively poor spatial resolution compared with MRI; however, to compensate for this, PET-CT or PET-MRI scans combining PET with CT or MRI are used to monitor clinical conditions.^95^ PET-MRI quantitative imaging of Aβ deposition delineated brain regions of amyloid vulnerability and traced regional pathology in an Alzheimer's disease mouse model.^96^ Additionally, the progression of cognitive impairment in the preclinical stage of Alzheimer's disease was predicted using Aβ PET-validated EEG-machine learning algorithm.^97^ Utilizing fluorine 18 fluorodeoxyglucose PET and dimensionality reduction techniques in patients with Alzheimer's disease, a computational model of Alzheimer's disease was proposed to map brain functions to neuroanatomy.^98^ These studies demonstrate the need to incorporate technology other than single PET imaging and propose ways to develop therapeutic strategies by building new models of brain diseases.

### EEG

EEG is an electrophysiological technique that quantifies microscopic electrical activity in the brain using electrodes attached to the scalp.^99^ The electrical activity generated by nerve cells is called a brain wave and is observed as a complex oscillating waveform.^99,100^ Brain waves are usually characterized by brain regions and frequency.^101^ EEG signals reflect brain status and are used to determine the depth of anaesthesia and diagnose various types of neuropsychological diseases such as epilepsy, encephalopathy, sleep disorders, coma and brain death.^100,102^ While other methods, including fMRI, single photon emission CT and PET, record brain activity indirectly through changes in blood flow or metabolic activity, the EEG has high temporal resolution by directly assessing brain electrical activity.^100^ However, compared with other imaging techniques, the EEG has a low spatial resolution because the brain consists of complex structures and tissues with different electrical conductivities.^103^ To compensate for these limitations, various attempts have been made to simultaneously measure and combine EEG and fMRI signals.^104^ EEG-fMRI signal coupling has identified individuals with significant cerebral amyloid deposition associated with cognitive impairment.^105^ Moreover, simultaneous EEG-fMRI data have enabled the identification of dynamic functional connectivity states associated with the epileptic brain.^106^ The utility of this method should be determined before commencing simultaneous EEG-fMRI recordings by considering the experimental design first, because combining EEG-fMRI does not always maximize the benefits.^104^

### Limitations of brain imaging approaches

The development of imaging technologies has contributed to the accuracy and efficiency of the diagnosis and treatment of brain diseases.^107,108^ Advances in these technologies allow early diagnosis and with the aim of improving the quality of life of patients.^107,108^ However, most cutting-edge imaging technologies, such as machine learning applications, are not yet widely used, and their application for the diagnosis and prognosis of specific brain diseases has only recently been introduced.^109^ In addition, the use of imaging technology raises important safety concerns.^110^ There is a risk of exposure to radiation or strong magnetic fields with side effects, including damage to healthy cells and the development of cancer.^110^ Moreover, the widespread use of these innovative technologies is limited by costs and the need to verify recent research results. Although the biological causes of brain abnormalities have been identified in many cases, accurate and consistent diagnostic markers for brain diseases have not been fully revealed due to the complex interactions of the CNS.^111^ Additionally, brain imaging requires the collection of considerable amounts of data to derive meaningful results and interpretations; however, the process of securing *in vivo* data using animal brains is time-consuming and labour-intensive.^112^ Considering these issues, predicting the prognosis of a specific disease based on imaging data alone is premature. Importantly, as in omics research, even with powerful technical tools that can acquire high-resolution brain imaging data, treatment options for most brain diseases have not yet been clearly identified. To overcome these difficulties, many researchers have attempted to create detailed molecular maps that integrate biomolecular information with imaging technology to develop effective biomarkers by building a database that serves as the basis for identifying brain diseases.^111^ The probability of finding clues for the treatment of brain diseases will increase with the accumulation of these data based on accurate locational and molecular information.

## Conventional integrative approaches for omics and brain imaging technology

### Recent trends

Over the past few decades, omics and brain imaging techniques have been used to decipher the complexity of brain dysfunction. Although neuropathology has been detected in omics and brain imaging datasets, little is known about practical solutions for alleviating severe symptoms of brain impairment. Based on the current understanding of neurodegenerative diseases, integrative approaches for imaging and omics data are emerging to develop potential diagnostic, therapeutic, and preventative strategies.^113^ To reveal the intermodal relationships among structural MRI, genome-wide association studies and transcriptomics in Alzheimer's disease, a novel combined model, Genotype-Expression-Imaging Data Integration, was presented through federated linear regression between structural MRI imaging biomarkers and gene expression for stratified populations with genome-wide association studies data, which surpasses expression quantitative trait loci methods for identifying genetic and transcriptomic factors involved in Alzheimer's disease.^113^ In another study focusing on multi-modality neuroimaging and genetic data such as MRI, PET and single nucleotide polymorphisms, each modality was combined using a three-stage deep feature learning and fusion framework for Alzheimer's disease diagnosis.^114^ In addition, a newly proposed machine learning framework showed potential associations between gene lists and Alzheimer's disease after evaluating human brain gene expression levels sourced from Accelerating Medicines Partnership-Alzheimer's Disease Datasets to identify candidates for drug repurposing in Alzheimer's disease.^115^ Moreover, a deep neural network architecture, Joining Omics and Imaging Networks via Graph Convolutional Layers and Attention, has been suggested for Parkinson's disease classification and has been used to model multi-omics data by integrating diffusion tensor imaging and fMRI data with genetic data.^116^ By combining blood metabolomics data with fluorodeoxyglucose- and fluorodopa-PET data, metabolic signatures were identified using diagnostic predictive performance in receiver operating characteristic analyses to detect Parkinson's disease-associated imbalances and to validate practical predictive models of Parkinson's disease.^117^ Overall, integrative approaches to omics and brain imaging technologies have the potential to greatly improve our understanding of complex neuropathology and lead to the development of innovative tools for therapeutic interventions.

### Limitations of conventional integrative approaches for omics and brain imaging technology

The integration of omics and imaging data has provided new opportunities for the diagnosis, treatment and prediction of brain diseases. However, unresolved challenges remain regarding the integration of omics and imaging. The first limitation is the small sample size, as a high-quality dataset with a large sample size is important for robust and reliable results in the fusion of information from multi-modality neuroimaging and omics data.^113,118^ The second issue is data heterogeneity in the process of integrating different data distributions and types after separate data pre-processing.^118^ Due to data heterogeneity, different omics and imaging data features may lead to inaccurate noise results.^114^ The third issue is the high dimensionality of features. As they do not share tactics to diminish data dimensionality, processing millions of images to acquire region of interest-based features does not downscale the dimensionality of omics data.^114^ To achieve full benefit of the revolutionary integration of omics and brain imaging technologies to identify new drug targets or biomarkers for brain diseases, further development of state-of-the-art techniques that address these challenges is required.

## Machine learning for data integration of multi-omics

### Basis of machine learning for multi-omics

Multi-omics data are commonly displayed as graphs or networks. For the integration of multi-omics data, recent studies suggested deep learning-based multi-omics integration methods using a transformer,^119-121^ an autoencoder^119,122-125^ and graph convolutional networks.^119,126,127^ They are based on the mathematical equations that have been used in deep learning.

Deep learning-based transformers are increasingly used to integrate heterogenous multi-omics data. Recent studies used transformer approaches for Cancer Classification,^120^ rare population inference,^121^ and complex disease classification.^119^ The transformer model is composed of an encoder and a decoder. The encoder consists of a multi-head self-attention layer and a feed-forward layer. The decoder consists of a feed-forward neural network, multi-head self-attention, and masked multi-head self-attention.

An autoencoder aims to reduce the dimensionality of high-dimensional multi-omics data^122^ and integrate heterogenous multi-omics data.^123-125^ Input data *x* in the original space is passed through the encoder *f* and compressed in latent space *z*.


z=f(x).

The decoder *g* recovers the compressed value *z* to the output data $x^{\prime }$ in the original space.


$$x^{\prime }=g(z)=g(h(x)).$$

The loss function *L* is calculated using the following equation:


$$L(x,x^{\prime })=\min(\|x-g\cdot f(x)\|)$$

graph convolutional networks aims to extract the most important information from the graph G=(V,E). A feature matrix X=N×D and an adjacency matrix *A* are assigned to the input values. The hidden layer of graph convolutional networks is determined by the non-linear function: $H^{\left(l+1\right)}=f(H^{\left(l\right)},\;A)$, where $H^{\left(0\right)}=X$ and $H^{\left(L\right)}=Z$. *L* indicates the number of layers. The propagation rule introduced by Kipf and Welling^128^ can be written as the following equation:


$$f(H^{\left(l\right)},\;A)=\sigma \left({\hat{D}}^{-\frac{1}{2}}\hat{A}{\hat{D}}^{-\frac{1}{2}}H^{\left(l\right)}W^{\left(l\right)}\right)$$

where *σ* is a non-linear activation function. $\hat{D}$ represents the diagonal node degree matrix, and ${\hat{D}}^{-\frac{1}{2}}\hat{A}{\hat{D}}^{-\frac{1}{2}}$ indicates a symmetric normalization. *W* is a weight matrix for the *l*th neural network layer.

The advancement of technology has enabled a more thorough analysis of biological networks, offering new insights into intricate biological processes such as cell differentiation and disease progression.^129^ The representation of these networks involves graphs or networks, where nodes stand for biological entities such as proteins, and edges indicate their interactions.^129^ The networks provide a basis for comprehending complex biological processes in depth. Deep learning, a potent form of neural network, is where the true value of this large dataset is being unlocked by capturing the non-linear relationships between different data features.^130^ The effectiveness of deep learning in deciphering complex patterns from noisy and intricate data has established it as a popular option in bioinformatics, a field where biological processes often involve numerous interacting components.

The use of deep learning in bioinformatics is optimized for graph structured data, facilitating a thorough examination of biological networks.^131^ The initial trial of applying deep learning in this area indicates promising results, often surpassing those achieved by traditional machine learning methods. These approaches utilize the unique structure of biological networks, harnessing the intrinsic relationships between components in the biological realm to extract valuable information that would otherwise be challenging to acquire.

The main goal of machine learning, especially deep learning, is to conduct multi-omics data analysis to identify patterns that conventional analytic methods may not catch. For example, machine learning is being used to integrate multi-omics data for research of brain diseases to overcome limited understanding about their pathophysiology and heterogeneity.^132,133^ Examining a range of omics data, including gene expression and methylation, is beneficial for elucidating the causes of brain diseases like Alzheimer's disease.^126^ This advanced technology could overcome these limitations and further suggest a breakthrough in the development of treatments by enabling a deeper understanding of the aetiology by integrating massive data from various fields.

Despite this, the challenges of high-dimensional low-sample size analysis and the complexity of biological processes demand the utilization of powerful numerical tools such as deep learning for precise analysis.^134^ Recently, machine learning techniques have been applied to the diagnosis of Alzheimer's disease, where deep learning is emerging as a solution for dealing with high-dimensional low-sample size data and making accurate predictions, particularly in gene expression datasets.

Therefore, integrating multi-omics data with deep learning may be the cornerstone for the diagnosis of brain diseases, personalized medicine and targeted therapies. Utilizing deep learning to comprehend the intrinsic connections in biological networks may be a game-changer that can contribute to the advancement of knowledge and the development of new therapeutic strategies.

## Explainability in machine learning

Machine learning and deep learning models have been utilized in various bioinformatics fields. These models provide key information extracted from biomedical databases for various purposes, including disease diagnosis and identification of drug targets by biological scientists. Although these models are beneficial to biologists, they are limited by a lack of interpretability and transparency, commonly called as a ‘black box’.^135^ A black box model is a system that does not disclose its internal mechanisms and cannot be understood through their parameters.^136^ Even if these patterns have high-accuracy prediction ability, their unclearness could be a source of mistrust because of the inability to grasp and validate the results. For a long time, machine learning models were universally considered as black boxes because scientists could not explain what happened to the data between the input and the output.^137^ Applying these black box models to highly susceptible fields such as healthcare or others related to human life, where moral and fairness issues have spontaneously occurred, is particularly challenging because of the limitation of trust.^138,139^ To overcome the limitations of the black box models, explainable artificial intelligence was developed to make deep learning technologies more understandable and transparent to human experts by interpreting how machine learning and deep learning models make decisions.^140^ Explainability in machine learning means that experts can describe what happens in the model from input to output. The more explainable the data, the deeper the understanding scientists get regarding the internal mechanisms that occur during training or decision-making.^139^ Explainable artificial intelligence helps to develop trust by reinforcing the stability, predictability, and repeatability of interpretable models.^137^ To discover and identify biomarkers in the biomedical field, handling a large-scale of data includes several obstacles such as high dimensionality, heterogeneity, noise and incompleteness, unstructured formats and high levels of uncertainty. Nevertheless, explainable artificial intelligence, with its high interpretability and explainability, enables scientists comprehend and handle the data easier.^140^

## Future direction (perspectives)

The combination of developments in imaging technology investigating the structure and function of the brain and omics, which analyses molecular information such as genes and proteins, has enormous potential for the treatment of brain diseases.^141^ However, technological progress in these research methods has not translated into clear diagnostic tests for brain diseases, such as Alzheimer's disease, Parkinson's disease, autism spectrum disorder and epilepsy. Rather than simply trying to improve existing experiments through technological advancements, there is a need to research new ideas for integrating different technologies to solve pathological brain problems.^40^

**Figure 1:**
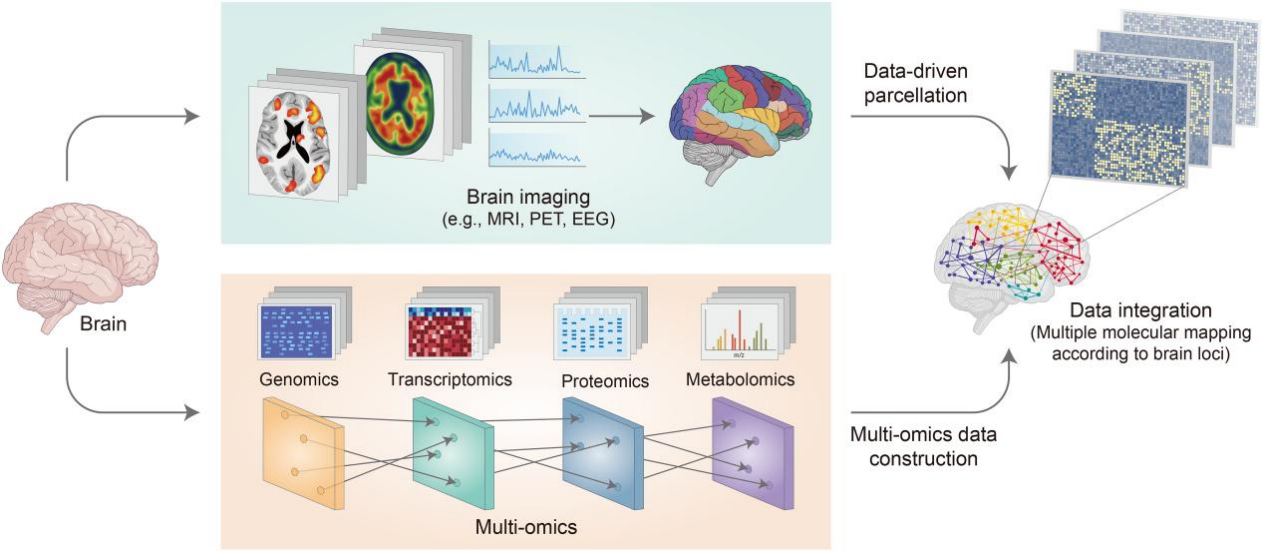
**Integrative approach of omics and imaging data for brain multi-molecular mapping**. Brain multi-molecular mapping reflecting the regional diversity of the brain can be accomplished by an integrative approach using data derived from microscale omics analysing biomolecules, as well as macroscale imaging technology capturing locational profiles. In the approach, a large amount of highly qualified data is obtained from omics and brain imaging technologies from the same brain sample. Omics addresses the multi-molecular level of distinct layers such as the genome, transcriptome, metabolome and proteome, and imaging technologies such as MRI, PET and EEG provide image profiles at a phenotypic level. The results can be processed through procedures including feature extraction using machine learning and then integrated to produce multi-molecular maps according to brain locus by detecting similar features. Data normalization can allow association between the microscale information on the actions of various molecules analysed using omics technologies with specific brain loci. MRI, magnetic resonance imaging; PET, positron emission tomography; EEG, electroencephalogram.

The brain is a complex system in which many cells interact with each other.^1^ Establishing a brain molecular map would greatly help in understanding the complexity and multifunctionality of the brain. For brain multi-molecular mapping reflecting the regional diversity of the brain, an integrative approach using data derived from microscale omics to analyse biomolecules and macroscale imaging technology to capture locational profiles is a powerful tool (Fig. 1). Using this approach, plenty of high-quality data can be obtained from omics and brain imaging technologies, which delineate different perspectives and types of information from the same sample. Omics addresses the multi-molecular levels of distinct layers, such as the genome, transcriptome, metabolome and proteome. Conversely, imaging technologies, such as MRI, PET and EEG, provide image profiles at the phenotypic level by examining tissues or tracking specific molecules in the brain.^118^ After securing each result, the results collected by the application of omics and imaging technologies can be processed through appropriate procedures, including feature extraction using machine learning, after which the data need to be integrated to produce multi-molecular maps according to the brain locus by detecting similar features.^118^ During this step, the global coordinate information of the mapping is presented using imaging features through data-driven brain parcellation that divides the brain into structural and functional parts to effectively understand its organization and function.^142,143^ Moreover, it is necessary to associate the microscale information on the actions of various molecules analysed using omics technologies with specific brain loci via data normalization.

**Figure 2:**
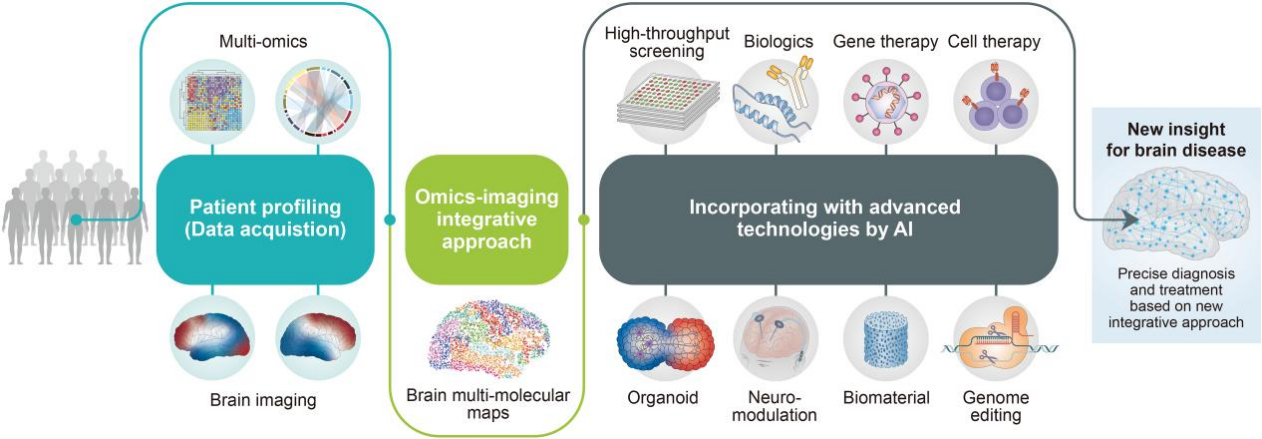
**Future direction for new insights into brain research through the utilization of omics-imaging integrative approach**. To apply the omics-imaging integrative approach in the future, the data acquisition step for patient profiling should involve gathering multiple data using multiple omics and brain imaging modalities from patients. Brain multi-molecular maps can be established using the omics-imaging integrative approach by correlating and combining omics and imaging data. Finally, incorporating an integrated molecular map with advanced technologies using artificial intelligence (AI) will help to establish new insights into brain diseases. AI, artificial intelligence.

One of the biggest issues in current brain disease research is that the accuracy of disease treatment or prediction is insufficient to significantly change people's quality of life.^2^ This study regards the omics-imaging integrative approach as important for addressing the actual burden caused by brain diseases and identifying the underlying pathological causes and therapeutic strategies. The purpose of this approach is to suggest future directions by utilizing an omics-imaging integrative approach to understand brain pathological systems (Fig. 2). To employ the omics-imaging integrative approach in the future, an initial step of data acquisition should be undertaken to gather data using multiple omics and brain imaging through the profiling of many patients. At this data acquisition step, it should be prior to establish reliable and highly reproducible omics and imaging analysis technology. And it is expected that many researchers will have to do fundamental work such as unifying the methodology and confirming reproducibility. Based on the collected patient database, the omics-imaging integrative approach can establish brain multi-molecular maps by correlating and combining omics and imaging data. In addition, incorporating the integrated molecular map with advanced technologies through AI will provide new insights at the molecular level into diagnosis and treatment that are more accurate than the currently available therapies.^144^

## Data Availability

Graphical Abstract was created with BioRender.com. Data sharing is not applicable to this article as no new data were created or analysed in this study.
